# A novel deep synthesis-based insider intrusion detection (DS-IID) model for malicious insiders and AI-generated threats

**DOI:** 10.1038/s41598-024-84673-w

**Published:** 2025-01-02

**Authors:** Hazem M. Kotb, Tarek Gaber, Salem AlJanah, Hossam M. Zawbaa, Mohammed Alkhathami

**Affiliations:** 1 The Institute of Cancer Research, 237 Fulham Road, London, SW3 6JB UK; 2 School of Science, Engineering, and Environment, University of Salford, Manchester, M5 4WT UK; 3 Faculty of Computers and Informatics, Suez Canal University, Ismailia, 41522 Egypt; 4 College of Computer and Information Sciences, Imam Mohammad Ibn Saud Islamic University (IMSIU), Riyadh, 11432 Saudi Arabia; 5 Department of Computer Science, The University of Manchester, M13 9PL Manchester, UK; 6 Faculty of Computers and Artificial Intelligence, Beni-Suef University, Beni-Suef, Egypt; 7 Applied Science Research Center, Applied Science Private University, Amman, Jordan

**Keywords:** Computational science, Computer science, Information technology

## Abstract

Insider threats pose a significant challenge to IT security, particularly with the rise of generative AI technologies, which can create convincing fake user profiles and mimic legitimate behaviors. Traditional intrusion detection systems struggle to differentiate between real and AI-generated activities, creating vulnerabilities in detecting malicious insiders. To address this challenge, this paper introduces a novel Deep Synthesis Insider Intrusion Detection (DS-IID) model. The model employs deep feature synthesis to automatically generate detailed user profiles from event data and utilizes binary deep learning for accurate threat identification. The DS-IID model addresses three key issues: it (i) detects malicious insiders using supervised learning, (ii) evaluates the effectiveness of generative algorithms in replicating real user profiles, and (iii) distinguishes between real and synthetic abnormal user profiles. To handle imbalanced data, the model uses on-the-fly weighted random sampling. Tested on the CERT insider threat dataset, the DS-IID achieved 97% accuracy and an AUC of 0.99. Moreover, the model demonstrates strong performance in differentiating real from AI-generated (synthetic) threats, achieving over 99% accuracy on optimally generated data. While primarily evaluated on synthetic datasets, the high accuracy of the DS-IID model suggests its potential as a valuable tool for real-world cybersecurity applications.

## Introduction

The escalating frequency of security incidents has compelled organizations to implement robust security measures, both physical (e.g., perimeter fences and surveillance cameras) and digital (e.g., firewalls and authentication mechanisms). Although these controls are primarily designed to thwart external threats, research indicates that insider threats-executed by malicious, negligent, or discontented personnel within an organization-pose a more significant risk, accounting for 79% of cybersecurity issues ^1^.

Insider threats originate from internal entities, such as employees or devices, within an organization ^2^. These entities can initiate threats both intentionally and unintentionally. Due to their direct access to information, networks, and systems ^3,4^, internal actors pose a significantly higher risk compared to external threats  ^4^. Furthermore, their status as trusted or semi-trusted individuals, based on their location and affiliation, grants them elevated access privileges. This allows them to circumvent standard access control measures undetected ^5^.

Intrusion Detection Systems (IDS) play a pivotal role in safeguarding organizational assets by monitoring both insider and outsider threats  ^3^. These systems utilize sophisticated mechanisms, including anomaly detection and behavior analysis  ^6^, to identify potentially malicious activities (including fake user profiles) that deviate from normal operational patterns ^7^. However, the advent of generative AI and high-quality synthetic data has introduced new complexities  ^8^. These technologies facilitate the creation of highly convincing fake user profiles, enabling both insiders and outsiders to impersonate legitimate users effectively and evade traditional detection systems  ^9^.

Although numerous machine learning models, e.g., ^10–19^, have employed sophisticated mechanisms, such as anomaly detection and behavioural analysis, to identify activities that deviate from normal operational patterns, none of these models have addressed the challenges posed by the advent of generative AI and high-quality synthetic data. This paper aims to address this significant gap by proposing a novel detection approach that considers these advanced technologies. To do this, the paper aims to achieve three objectives:

1. Identify malicious internal users to detect potential security incidents at an early stage.
2. Evaluate the generative algorithms ability to mimic real user profiles.
3. Differentiate between real and AI-generated abnormal user profiles.

A novel Deep Synthesis Insider Intrusion Detection (DS-IID) method based on deep feature synthesis, generative models, and deep learning is proposed to accomplish these objectives. The deep feature synthesis is used to construct detailed tabular user profiles from event data, the generative algorithms are used to replicate real user profiles, and a binary deep learning model is used for classification. DS-IID not only aims to bridge conventional security gaps but also addresses the sophisticated challenges posed by synthetic data created by generative AI. The main contributions of the paper are:

1. Proposing a novel insider attack detection model (DS-IID) to address threats posed by generative AI, in addition to traditional ones. In addition to intrusion detection, the DS-IID model differentiates between real and AI-generated profiles.
2. Unlike previous work, the DS-IID uses the complete CERT dataset, including the three distinct abnormal scenarios. This allows for a more generalized and robust detection model.
3. The DS-IID model employs deep feature synthesis (DFS) to automatically generate detailed user profiles from raw event data. This reduces the need for manual intervention, making the model adaptable to various datasets and scenarios.
4. Providing a thorough evaluation of the DS-IID model using nine metrics: Cohen’s kappa, TPR, FPR, FAR, Recall, Precision, F1 score, Accuracy, and AUC. This comprehensive evaluation demonstrates the robustness and the ability of the model to maintain high performance while ensuring it is balanced in terms of false positives and false negatives, which is crucial for practical deployment in cybersecurity environments.

The rest of the paper is structured as follows. Section Related Work discusses existing insider threat detection systems. Section Materials and methods introduces the dataset, feature extraction and selection methods, and machine learning algorithms used to build the DS-IID intrusion detection method. Section Results and discussion presents the experiments and analyzes experimental results. Section Discussion compares the results to other studies demonstrating the potential of the proposed model. Finally, Section Conclusions concludes this paper.

## Related work

Insider threat intrusion detection systems can be classified into two categories: (i) generic intrusion detection systems and (ii) intrusion detction systems designed specifically to address data imbalances. A summary of these systems is presented in Table 1.

### Generic insider threat intrusion detection systems

Several IDS have been proposed to detect insider threats. Kim et al. ^10^ proposed a method that utilizes topic modelling and univariate Gaussian distribution to extract and select features, respectively. Once important features are identified, the method uses Gaussian density estimation, Parzen window density estimation, principal component analysis and K-means clustering algorithm to detect malicious insiders. To assess their method, experiments were carried out on CERT (Computer Emergency and Response Team) dataset, a publicly available dataset for insider threats. Depending on the amount of suspicious behaviours being monitored, experimental results show that the method can detect $54\%$ to $90\%$ of malicious insider threats. Al-Mhiqani et al. ^11^ proposed a Gated Recurrent Unit Neural Network (GRU) based method to detect insider threats. Experiments show that the method can provide up to $92\%$ accuracy and $29\%$ loss value when applied to the CERT dataset.

Le and Heywood ^13^ proposed a method that combines four unsupervised learning algorithms, AE (Autoencoder), IF (Isolation Forest), LODA (Lightweight On-Line Anomaly Detection) and LOF (Local Outlier Factor), to detect unlabelled insider threats. Experimental results show that the method provides a $90\%$ and $98\%$ AUC (Area Under the Curve) value when applied to CERT R4.2 and CERT R6.2 data, respectively. Pantelidis et al. ^14^ proposed a method that employs AE and VAE (Variational Autoencoder) deep learning algorithms to automate insider threat detection. Experimental results show that the VAE algorithm outperforms the AE algorithm in terms of accuracy by $1\%$. When applied to the CERT dataset, the AE and VAE algorithm provide $95\%$ and $96\%$ accuracy, respectively.

### Imbalanced datasets

Imbalanced datasets are a major concern in machine learning, as if they were used to train a model, the model will have a bias that affects its accuracy. To address this issue, Sheykhkanloo and Hall ^12^ proposed a spread subsample-based method to detect malicious behaviours in imbalanced datasets. The method uses the spread subsample technique (where a random subset of the data is selected after the maximum spread between existing classes is specified) to balance the dataset. The J48 Decision Tree (DT) algorithm, Support Vector Mechanism (SVM), Naïve Byes (NB) and Random Forest (RF) algorithm were then applied to ZoneFox2017 dataset to experimentally evaluate the method. The results show that applying the spread subsample technique did not improve the classifiers performance metrics (e.g., Classification Accuracy (CA) and True Positive (TP) rate), however, it reduced the time needed to build and test their models.

Al-Mhiqani et al.^15^ proposed a method, named AD-DNN, that combines Adaptive Synthetic Technique (ADASYN) and Deep Neural Network (DNN) to detect insider threats. The ADASYN algorithm is used to balance the dataset whereas the DNN classifier is used to detect threats. Although ADASYN is used to generate synthetic data to address data imbalances, the model does not distinguish between real and synthetic profiles. Experimental results show that the AD-DNN method provides $96\%$ accuracy and $95\%$ AUC value when applied to the CERT dataset. Sarhan and Altwaijry^16^ proposed a DFS (Deep Feature Synthesis) and PCA (Principal Component Analysis) based method. The DFS algorithm is used to extract features whereas the PCA is used to reduce dimensionality (i.e., the number of features). To evaluate the method performance, four classifiers (NN, SVM, AdaBoost, and RF) were tested on the CERT dataset. Evaluation results show that the method provides $91\%$ accuracy for anomaly detection and $97\%\sim 100\%$ accuracy for classification. Al-shehari et al.^17^ proposed an IF-based method to mitigate data imbalances and detect malicious behaviours. Depending on the dataset contamination ratio (i.e., the percentage of outliers in the dataset), experimental results show that the method provides $40\%\sim 96\%$ accuracy and detection rate when applied to the CERT dataset. Boppana and Bagade^18^ proposed a GAN (Generative Adversarial Network) based autoencoder, named GAN-AE, to detect unknown intrusions. The GAN is used to train the autoencoder while the autoencoder is used to detect anomalous network traffic in IoT (Internet of Things) applications. Experimental results show that GAN-AE provides 0.97 F1-score value when applied to FBMA (Flow-Based-MQTT-Attack) dataset.

Mouyart et al.^19^ proposed a method that combines CTGAN (Conditional Tabular Generative Adversarial Network), TPE (Tree-structured Parzen Estimator), and AE-RL (Adversarial Environment Reinforcement Learning) algorithm for insider threat detection. The CTGAN is used to generate new insider threats using deep learning while the TPE is used to optimize CTGAN performance. Once the synthetic threats are generated, they are combined with existing insider threats to balance the dataset. The AE-RL algorithm is then used to detect intrusions. Experimental results show that the method provides 0.0463 and 0.7617 F1-score values for unbalanced and balanced cases, respectively, when applied to the CERT dataset. It is worth noting that although Mouyart et al. used CTGAN to generate synthetic insider threats, the model ability to differentiate synthetic threats from real ones has not been discussed in the paper.

**Table 1:** A summary of recent insider threat intrusion detection systems

Ref No.	Year	User profiles or event classification	Feature extraction techniques	Classification method	Classifier(s)	Dataset	Discriminating synthetic (AI-Generated) and real malicious users	Results
^10^	2019	Event classification	TM	One-class classification	Gauss, Parzen, PCA, KMC	CERT	No	Accuracy= at most 98.67%
^11^	2020	Event classification	-	Binary classifier	GRU	CERT	No	Accuracy = 92% Loss value = around 0.29
^12^	2020	Event classification	-	Binary classifier	J48 DT, SVM, NB, RF	ZoneFox	No	Accuracy on balanced datasets= at most 98% Accuracy on imbalanced datasets= at most 99%
^13^	2021	Event classification	Random	UL	AE, IF, LODA, LOF	CERT	No	Detection rate = 77% to nearly 100%, when 1% and 5% of the most suspicious instances are investigated, respectively
^14^	2021	Event classification	-	Binary classifier	AE, VAE	CERT	No	AE results: Accuracy= 95%, Precision= 90% Recall = 95%, F1 score= 92% VAE results: Accuracy= 96%, Precision= 92% Recall = 96%, F1 score= 94%
^15^	2021	Event classification	-	Binary classifier	ADASYN, DNN	CERT	No	Accuracy= 96%, F1 score= 95% AUC= 95%, FPR= 4% FNR= 5%, TNR= 96%
^16^	2023	Profile classification	PCA, DFS	Binary classifier	NN, SVM, AdaBoost, RF	CERT	No	Precision= 100%, Recall= 100% F1 score=100%, Accuracy= 100%
^17^	2023	Event classification	-	Binary classifier	IF	CERT	No	Accuracy= 40% to 96% F1 score= 58% to 99% Detection rate= 40% to 96%
^18^	2023	Event classification	Flow-based features	UL	AE	MQTT-IoT, FBMA	No	MQTT-IoT dataset results: Accuracy= 0.969, Precision= 0.971 Recall = 0.969, F1 score= 0.969 FBMA dataset results: Accuracy= 0.976, Precision= 0.977 Recall = 0.976, F1 score= 0.976
^19^	2023	Event classification	CTGAN, TPE	Multi-class & binary classifier	AE-RL	CERT	No	Unbalanced dataset results: F1 score= 0.046, Precision= 0.023, Recall = 0.966 Balanced dataset results: F1 score= 0.761, Precision= 0.682, Recall = 0.861

SL: Supervised learning; UL: Unsupervised learning; TM: Topic modeling; UGD: Univariate Gaussian distribution; Gauss: Gaussian density estimation; Parzen: Parzen window density estimation; PCA: Principal component analysis; KMC: K-means clustering; GRU: Gated Recurrent Unit Neural Network; DT: Decision Tree; SVM: Support Vector Mechanism; NB: Naïve Byes; RF: Random Forest; AE: Autoencoder; VAE: Variational Autoencoder; IF: Isolation Forest; LODA: Lightweight On-Line Anomaly Detection; LOF: Local Outlier Factor; ADASYN: Adaptive Synthetic Technique; DNN: Deep Neural Network; DFS: Deep Feature Synthesis; GAN: Generative Adversarial Networks; CTGAN: Conditional Tabular Generative Adversarial Network; AE-RL: Adversarial Environment Reinforcement Learning; TPE: Tree-Structured Parzen Estimator; CERT: Computer Emergency and Response Team; FBMA:Flow-Based-MQTT-Attack-Dataset

### Gap in knowledge

The related work discussed earlier (and summarized in Table 1) shows that various methods such as GRU-based models, Gaussian Density Estimation, Autoencoders, and deep learning techniques like VAE and AE have proven effective in identifying traditional insider threats, especially on datasets like CERT. For example, VAEs can achieve high levels of accuracy, even reaching 96% in detecting known patterns. However, while these models perform well on static or predefined data, they fall short in handling the more dynamic, evolving threats that could be introduced by generative AI in real-time. Generative AI allows attackers to create synthetic behaviors or profiles that closely mimic legitimate users, making detection much more difficult. The current literature does not respond to these kinds of new patterns because they rely heavily on predefined data and static detection mechanisms. By relying on historical datasets and methods tailored to known threats, these approaches leave a significant gap when faced with generative AI. The challenge is that these methods are not designed to learn from evolving data in real time or deal with adversarial tactics that produce synthetic threats at scale. This challenge will be addressed in this paper by proposing the DS-IID model. By using techniques like CTGAN and Gaussian Copula, the DS-IID does not just work with historical data-it actually learns from evolving patterns and adapts in real-time. With its integration of generative models and deep learning, DS-IID offers a more adaptive solution to tackle the growing challenges posed by AI-driven threats.

## Materials and methods

This study provides an advanced DS-IID system to detect insider threats by leveraging modern machine learning algorithms, specifically deep learning, and applying them to tabular data. The meticulous preparation of the dataset is crucial in implementing any machine learning algorithm effectively. Therefore, it is imperative to thoroughly preprocess raw data and extract features relevant to the learning task of identifying malicious users contributing to abnormal events or those potentially characterized as synthetic users.

### Dataset and feature extraction

A dataset is a collection of examples or instances used in machine learning to train, validate, and test models ^20^. The dataset is typically divided into two or three subsets: the training set, used in training the model; the validation set, used to tune parameters and avoid overfitting; and the test set, used to evaluate the model’s performance on unseen data. Datasets can be structured or unstructured and may include various types of data, such as numerical, categorical, and text. Feature extraction involves identifying or transforming relevant information from raw data to produce features suitable for utilization as input for machine learning models. In the context of structured data, feature extraction involves selecting relevant columns or transforming existing columns. Effective feature extraction is essential for building accurate and efficient machine learning models, as it helps the model focus on relevant patterns and relationships within the data  ^20^. It is often a critical step in the preprocessing phase before training a machine learning model.

#### Dataset

Researchers face a significant challenge in studying insider threats due to a lack of actual data, particularly log files containing private user information ^21^. Organizations often restrict access to real data to protect their users and assets. However, some organizations may grant researchers limited access to anonymized data under specific regulations. This obstacle hinders researchers from effectively addressing the insider threat detection problem. To overcome this challenge, it is preferable to use synthetic data in designing and evaluating detection systems. While datasets such as DARPA ADAMS  ^22^ and Schonlau  ^23^ have been used in previous research, they are less suitable for the complex insider threat problem.

Over the past decade, the CMU-CERT dataset, created by the Community Emergency Response Team at Carnegie Mellon University  ^24^, has become widely utilized for insider threat detection systems  ^25^. The CERT insider threat dataset has seen multiple releases, with versions r4.2 and r6.2 being the most commonly used. Notably, CERT r4.2 exhibits a higher rate of malicious activities compared to others  ^16^.

Our focus in this study is on the CERT r4.2 dataset, consisting of logon/logoff events, email transmissions, device usage, file activities, and HTTP events involving 1000 employees over 17 months, 930 can be categorized as normal users and the other 70 are users who have been involved in malicious activities. The dataset includes 32,770,222 events from both normal and abnormal users, intentionally incorporating 7323 instances of malicious insider activities. Each employee’s psychometric score, reflecting the “Big Five personality characteristics”, is also included in the dataset.

The insider threats in CERT r4.2 are categorized into three primary scenarios:

1. An individual who had never engaged in after-hours work or utilized removable drives has recently started logging in after hours, employing a removable drive to upload information to wikileaks.org, and subsequently departed from the company shortly thereafter.
2. A person exploring job opportunities on career websites and reaching out to potential employers is observed taking data at an accelerated rate using a thumb drive before leaving the office, deviating from their typical actions.
3. A discontented system administrator installs a keylogger and transfers it to the computer of his supervisor via a thumb drive. The following day, he accesses the company’s network masquerading as his boss, disseminating an alarming mass email that triggers widespread concern before promptly exiting the organization.

In this study, our analysis considers all three scenarios, and hence the relevant data were extracted from all files.

#### Deep feature synthesis

Machine learning algorithms heavily depend on the selection of input features, making it a crucial aspect of algorithmic design. While many machine learning algorithms necessitate a thoughtful and intuitive feature selection process, recent advancements in deep learning have allowed for the automatic learning of features through the network architecture. Despite this, feature selection remains a time-consuming phase in many machine learning tasks, guided by human intuition.

This study leverages automated feature engineering to enhance the efficacy of insider threat detection approaches. The Deep Feature Synthesis (DFS) tool  ^26^ goes beyond manual feature selection by automatically generating features for relational datasets. It utilizes a novel Gaussian Copula approach to perform feature engineering on different tables and transactional information in databases and log files. Thus, DFS is well-suited for generalizable machine-learning pipelines. It considers both previously selected features and introduces additional features, resulting in a more comprehensive set. Additionally, it allows data scientists to save time through the consistent application of mathematical aggregation and transformation functions. Consequently, it eliminates the need to manually aggregate data using various statistical functions.

Despite its automated nature, the DFS algorithm captures features that benefit from human interpretation. The features generated by DFS are not only time-efficient but also easily understandable. This is attributed to their foundation in primitive combinations that can be described in natural language. This aids data scientists in comprehending the features created by DFS.

As inputs, the DFS algorithm takes entity sets, relationships between entities, and mathematical functions to be applied during feature extraction. Entity sets can have diverse data types, including numeric, categorical, timestamps, and free text. We processed and used each file in the CERT dataset as an entity set.

**Fig. 1:**
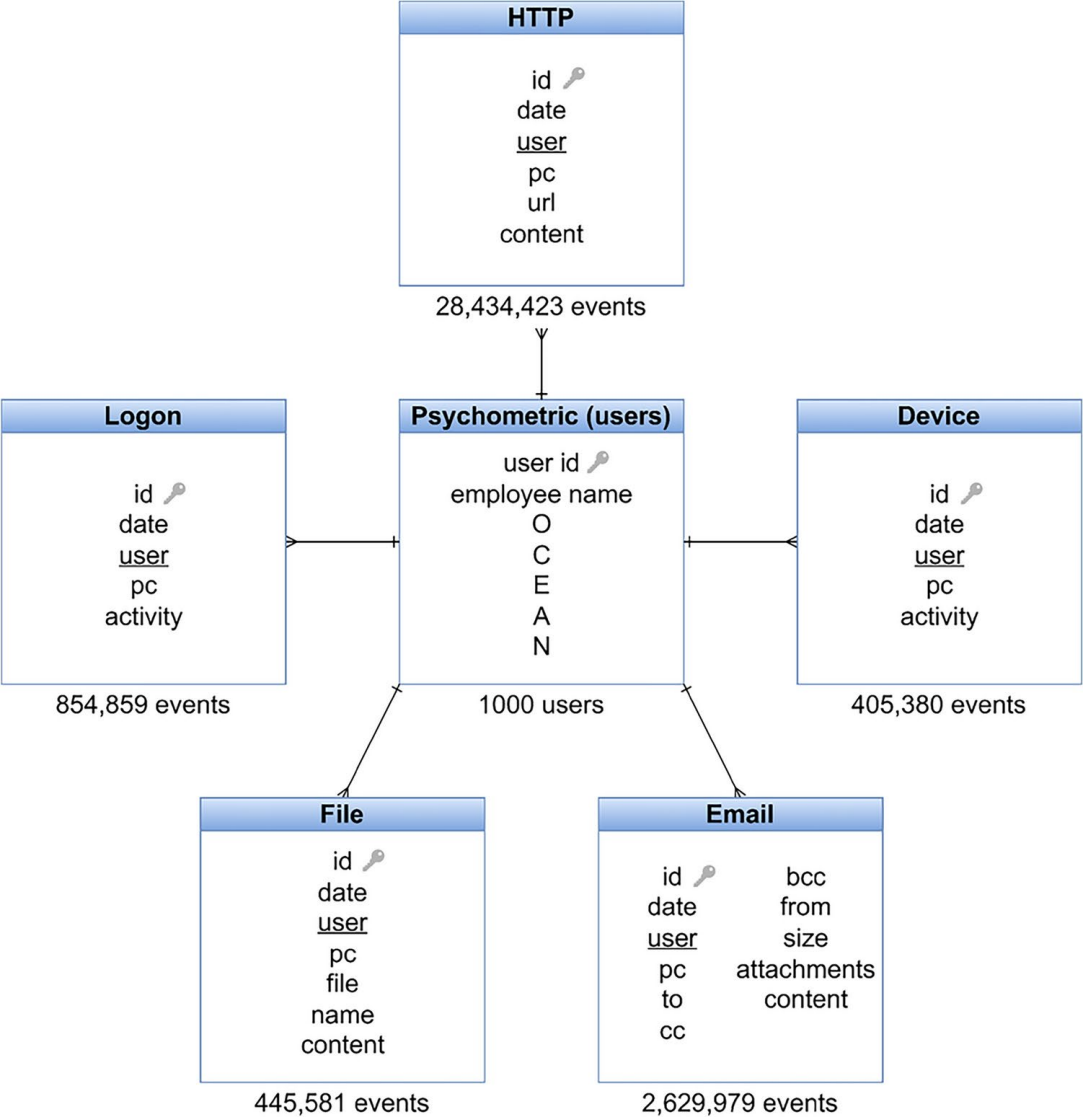
A simplified schema of the CERT dataset. There are 6 entities. An arrow from one entity to another signifies that the first entity references the second in the dataset. The schema shows the many-to-one relationship between all the entities and the Psychometric entity. A key sign denotes the primary key in each entity, and foreign keys are underlined.

DFS allows two main types of relationships between entities: i) A forward relationship between an instance of one entity and a single instance of another entity. Forward relationships are used to transfer features from one entity (the second) to another (the first). ii) A backward relationship from an instance of one entity to all instances of another entity that have a forward relationship to the first entity. Backward relationships are used to aggregate information from related entities to generate new features for the original entity. These two relationships play a crucial role in feature generation and extraction, allowing for the creation of complex and informative features based on the connections between different entities. In the CERT dataset, each event in a file is attached to a user. Therefore, all the entities in the CERT dataset reference the psychometric entity (the user entity), as presented in Figure  1.

DFS generates three types of features: Entity, Direct, and Relational features. Entity features involve computations at the entity level, such as transforming existing features or converting data types. Direct features are applied over a forward relationship between two entities. An example is adding the psychometric scores to the logon events entity. However, in mapping the CERT dataset, our target is generating user profiles. Therefore, the forward features are not utilized. On the other hand, relational features are calculated over backward relationships by aggregation functions (such as SUM, MEAN, MAX, MIN). For example, calculating the total size of email attachments for each user by summing the attachment sizes related to each user. Thus, backward features come in handy during the feature extraction process in the CERT dataset. The number of recursive steps or levels of feature generation from the base field to the final feature determines the depth in DFS. The algorithm tracks relationships in the data to a base field, employing mathematical functions along that pathway to generate the final feature. By stacking calculations sequentially, each new feature is defined to have a certain depth. We set the maximum feature depth to two and applied all compatible functions from the available collection of DFS aggregation primitives and compatible transform functions based on the default DFS transform primitives (Table 2).

**Table 2:** Lists of the used aggregation and transform primitives during the process of feature extraction with Deep Feature Synthesis

Aggregation primitives	Transform primitives
percent unique, time since first, variance, num true, time since last min, last, count greater than, correlation, num peaks, first last time delta, count outside range, avg time between, time since last false, date first event, percent true, std, count, first, count outside nth std, mode, n most common, any, median, average count per unique, time since last true, entropy, num true since last false, min count, max consecutive negatives, num consecutive less mean, num false since last true, kurtosis, max consecutive positives, sum, count less than, count below mean, auto correlation, max consecutive true, min, num consecutive greater mean, time since last max, trend, all, max consecutive zeros, time since last, mean, num zero crossings, num unique, max, max consecutive false, skew, count inside nth std, count inside range, count above mean	day, year, month, weekday, num words, num characters, hour

### Model building for tabular data

Deep learning, a paradigm that has revolutionized various domains of artificial intelligence, has historically found its stronghold in tasks such as image and speech recognition ^27,28^. However, when it comes to tabular data - structured information organized in rows and columns - traditional machine learning models, e.g., decision trees and gradient boosting, have been the conventional choices ^29^. Deep learning’s penetration into the realm of tabular data has been relatively less common, largely owing to the interpretability of traditional models and the need for substantial amounts of data for deep models to excel.

Despite its limited prevalence in tabular applications, the landscape is evolving. Researchers and practitioners are actively exploring ways to harness the power of deep learning for structured data. As the integration of deep learning with tabular data is still an evolving area, there is a need to adapt existing architectures to suit the unique characteristics of structured datasets. We delve into the adaptation of simple neural architecture for DFS tabular features, exploring the potential benefits of applying deep learning to structured information.

#### Proposed insider attack detection model (DS-IID)

**Fig. 2:**
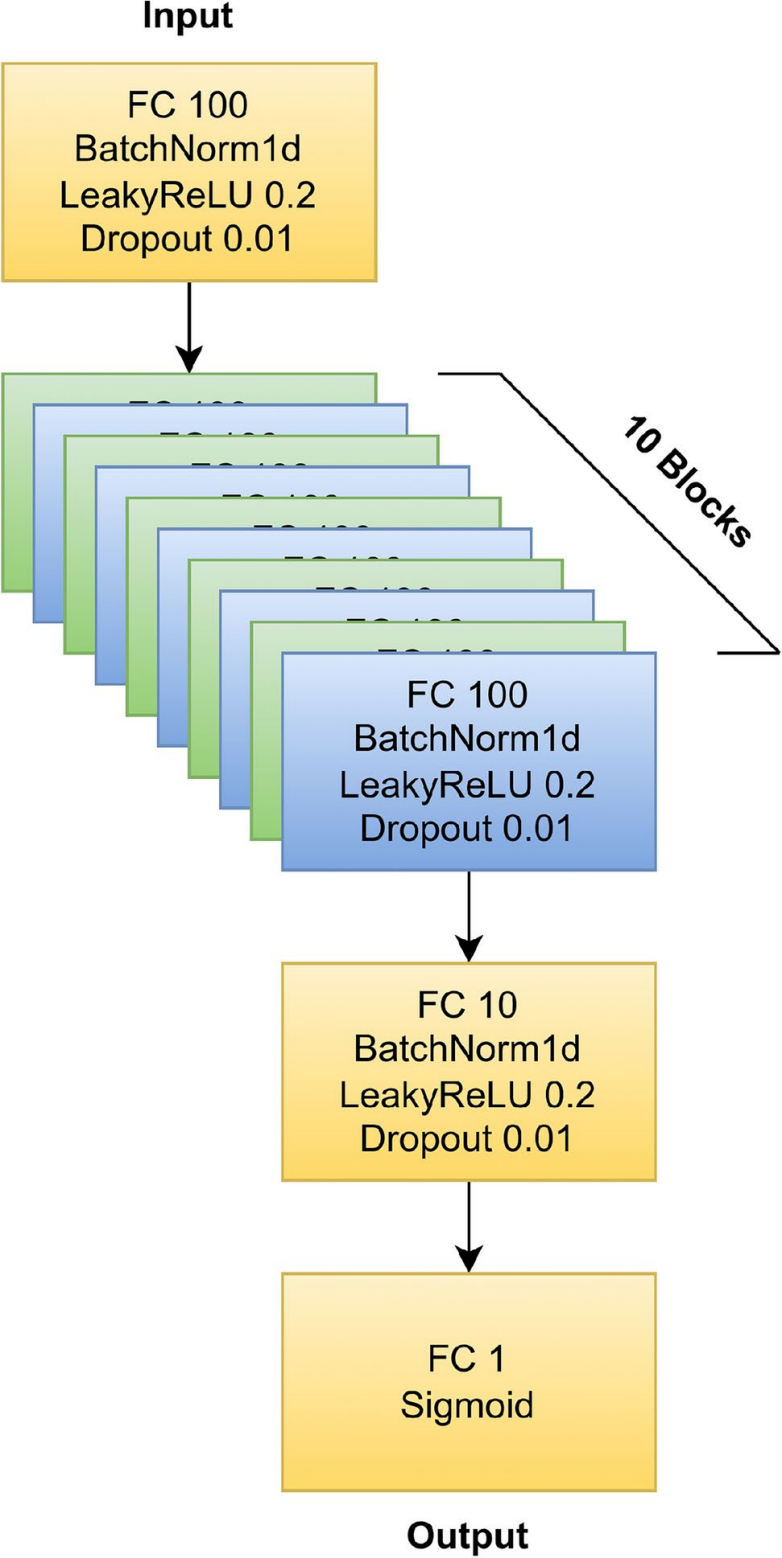
The neural network architecture diagram. It consists of an input block followed by ten hidden blocks. Each block includes a linear fully connected layer with 100 neurons, Batch Normalization, Leaky ReLU activation, and Dropout. The architecture ends with one more hidden block with ten neurons and an output layer with one neuron

The neural network architecture employed in this study is crafted using fundamental building blocks, where each block consists of a linear fully connected layer, Batch Normalization, Leaky ReLU activation, and Dropout. The initial block comprises an input layer with 100 neurons. Subsequently, ten identical blocks are sequentially added, each consisting of 100 neurons. The architecture culminates in a block with 10 neurons, followed by a fully connected layer with a single neuron and a Sigmoid activation function. This design indicates its applicability to binary classification tasks. All these layers are organized sequentially within the model as shown in Figure 2.

To ensure robust training, the weights of the linear layers in the model undergo initialization using the Kaiming normal initialization method ^30^. In this method, the weights are initially randomly sampled from a Gaussian probability distribution with a mean of 0.0 and a standard deviation of sqrt(2/n), where n is the number of inputs to the node. The Kaiming initialization method is specifically tailored to accommodate the leaky ReLU nonlinearity, contributing to the network’s capacity to effectively learn and generalize from the input data during the training process.

#### Model training

The training process of the neural network involves several key steps, and preparing the data for training involves various preprocessing steps. The dataset is shuffled, and then it is split into training, validation, and test sets. Feature scaling is performed to standardize the input features. Additionally, the class distribution is calculated, and upsampling weights are determined for each class to address potential class imbalance. The data loading process is customized to create balanced batches for training using the calculated class distribution. The loss function is defined as binary cross-entropy loss. The Adam optimizer is employed, and a learning rate scheduler is used to dynamically adjust the learning rate during training. A warm-up scheduler is implemented to gradually increase the learning rate during the initial epochs, then reduce learning rate on the plateau. The model is trained on batches of data with a batch size of 16. The training process includes forward and backward passes, parameter updates, and logging of training statistics. The training loop also evaluates the model on the validation set, calculating validation loss and accuracy. Early stopping is applied to prevent overfitting.

### Discriminating normal and abnormal users

As the world becomes increasingly digital, the security of corporate systems and applications is of utmost importance. One of the ways to ensure the safety of their platforms is by detecting abnormal user profiles. This can be achieved using neural networks. The built neural architecture is trained to discriminate normal and abnormal user profiles to ensure the security of the platforms. Neural networks are a powerful tool that can detect patterns in datasets. It can analyze user data and identify normal and abnormal user profiles based on the patterns in their behavior.

### Producing synthetic user profiles

Synthetic user profiles are fabricated identities created to mimic legitimate users within a corporate environment. These profiles may include false credentials, employment history, and other attributes that make them appear authentic. Unlike traditional insider threats, where a disgruntled employee may intentionally engage in malicious activities, the individuals behind synthetic profiles often operate covertly with the aim of remaining undetected.

The use of artificial intelligence (AI) to generate synthetic data has become a critical tool in various domains, offering unique advantages in data-driven research, privacy preservation, and model development. While AI-generated synthetic data offers numerous benefits, there is a potential for misuse, particularly in the realm of cybersecurity. Cyber attackers may exploit the technology to create synthetic user profiles for malicious purposes. Synthetic profiles can be used to generate fictitious accounts or impersonate legitimate users, leading to unauthorized access, data breaches, and other cyber threats.

Advancements in AI have significantly elevated the quality of generated data. In the realm of text data, Natural Language Processing (NLP) models have exhibited remarkable proficiency in understanding and generating human-like text. These models are instrumental in tasks such as content creation, language translation, and even code generation, demonstrating the evolving capabilities of AI in the textual domain. Images are generated with unprecedented accuracy, primarily due to the advent of Generative Adversarial Networks (GANs). Tabular data, a cornerstone of traditional data analysis, has not been left untouched by AI’s progress. The Synthetic Data Vault (SDV) is an extensive Python library designed to streamline the generation of synthetic tabular data ^31^. It offers a robust solution for users aiming to produce realistic, privacy-compliant tabular datasets across various applications. Utilizing sophisticated modelling techniques, SDV crafts data that mirrors the statistical characteristics of actual datasets, thereby ensuring the synthetic data’s relevance and security for research and development projects. Here, we use different AI algorithms implemented in SDV to produce tabular synthetic data similar to real user profiles. These algorithms can be categorized into i) Classical Machine Learning algorithms, ii) Deep Learning algorithms, and iii) Hybrid ML algorithms.

#### Classical machine learning algorithms

Classical Machine Learning (ML) algorithms play a crucial role in extracting meaningful patterns and insights from data. These algorithms are foundational in the field of artificial intelligence and are employed across various domains, from finance to healthcare. When it comes to data synthesis, classical ML algorithms can be used to generate synthetic data.

The Gaussian Copula is a statistical concept used in modeling the dependence structure between random variables. It is particularly useful in finance, risk management, and actuarial science for modeling the joint distribution of multiple variables. In the context of tabular data synthesis, the Gaussian Copula is utilized to model the relationships between columns in a database table  ^31^. By converting the original data distributions to standard normal distributions, the Gaussian Copula allows for the estimation of covariances between variables without being influenced by the specific shapes of the original distributions. This approach helps in building generative models for tables in relational databases, enabling the creation of synthetic data that preserves the statistical dependencies present in the original data. The Gaussian Copula facilitates the estimation of covariances and conditional parameters between related data points, which are essential for generating realistic synthetic data that captures the underlying structure of the original dataset.

#### Deep learning algorithms

Deep Learning (DL) algorithms play a crucial role in data synthesis. They excel in data synthesis tasks due to their ability to capture complex patterns and relationships within the data.

- GAN-based method Conditional Tabular Generative Adversarial Network (CTGAN) is a specific type of generative adversarial network (GAN) designed for generating synthetic tabular data. CTGAN is a state-of-the-art method used in the field of synthetic data generation, particularly for structured data such as relational databases. CTGAN works by training a generator model to create synthetic data samples that are similar to the original dataset while also training a discriminator model to distinguish between real and synthetic data. Through this adversarial training process, CTGAN learns the underlying data distribution and dependencies present in the original dataset, allowing it to generate realistic synthetic data that preserves the statistical characteristics of the real data. CTGAN can be used as part of the generative modeling process to create synthetic data for relational databases that closely resembles the original dataset.
- VAE-based method Tabular Variational Autoencoder (TVAE) is a variational autoencoder (VAE) designed specifically for generating synthetic tabular data. VAEs are generative models that learn the underlying structure of data and can be used to generate new samples that resemble the original data distribution. TVAEs are used to encode the features of tabular data into a lower-dimensional latent space and then decode them back to generate synthetic data samples. By training the TVAE model on a dataset, it learns the complex patterns and relationships present in the data, allowing it to generate new data points that are statistically similar to the original dataset. TVAEs are particularly useful for generating synthetic tabular data because they can capture the dependencies and correlations between different columns in a structured dataset.

#### Hybrid ML algorithms

Copula GAN is a novel approach that combines copula theory with GANs for generating synthetic data. Copula GAN leverages copulas to capture the complex dependencies and correlations present in the data, while GANs are used to generate realistic synthetic samples. By decoupling the marginal distributions from the dependence structure, Copula GAN can capture the intricate relationships between variables in the data, leading to accurate synthetic data generation. Copula GANs have shown promise in various applications where capturing the joint distribution of variables accurately is crucial. By combining copula theory with the power of GANs, Copula GANs offer a sophisticated approach to generate synthetic data that preserves the statistical properties and relationships of the original dataset.

### Discriminating synthetic and real user profiles

Insider threats pose a significant risk to organizations, with malicious insiders potentially causing severe damage by exploiting their privileged access. Synthetic user profiles, created with the intent to mimic genuine users, further complicate the task of identifying potential threats. Attackers could use realistic-looking synthetic profiles to infiltrate systems, launch phishing attacks, or manipulate online interactions. Traditional security measures may fall short in detecting these sophisticated attacks, necessitating the adoption of models specifically trained for these tasks. Therefore, the built neural architecture is trained to discriminate real and synthetic user profiles and evaluated to ensure the security of the platforms.

## Results and discussion

Insider threats have been extensively studied previously. In this research, we employ deep feature synthesis to generate informative features from relational tabular data capturing user actions and events. These features are utilized to train a deep learning model to distinguish between normal and abnormal user profiles. We further extend our study by applying various machine learning (ML) and deep learning (DL) methods to create synthetic user profiles. Additionally, we leverage the deep learning model to differentiate between synthetic and real users. This allows us to ascertain whether abnormal events are associated with authentic or fictitious users.

### Dataset and feature extraction

After mapping the dataset tables to entities and establishing relationships among them for deep feature synthesis, we generated features representing user profiles based on user actions from the dataset. Features with null or string values were excluded, resulting in a total of 430 features (see Table S1). This process refined the dataset for more effective subsequent processes, ensuring that the resulting features are quantitatively analyzable and relevant for further analysis or modelling.

### Model building for tabular data

The model was developed using PyTorch, as detailed in the GitHub repository (https://github.com/hkotb/insider-threats). It is designed to process an input comprising 430 features. These features are initially passed through an input block, followed by a sequence of 10 identical blocks that systematically process the data. After traversing these blocks, the data is directed through several final layers. These layers are structured to progressively reduce the dimensionality of the data, ultimately funnelling it down to a single neuron. This neuron constitutes the output layer, which is specifically configured for binary classification tasks. The architecture culminates in a streamlined model that contains 147,341 trainable parameters, as documented in Table S2.

### Discriminating normal and abnormal users

**Fig. 3:**
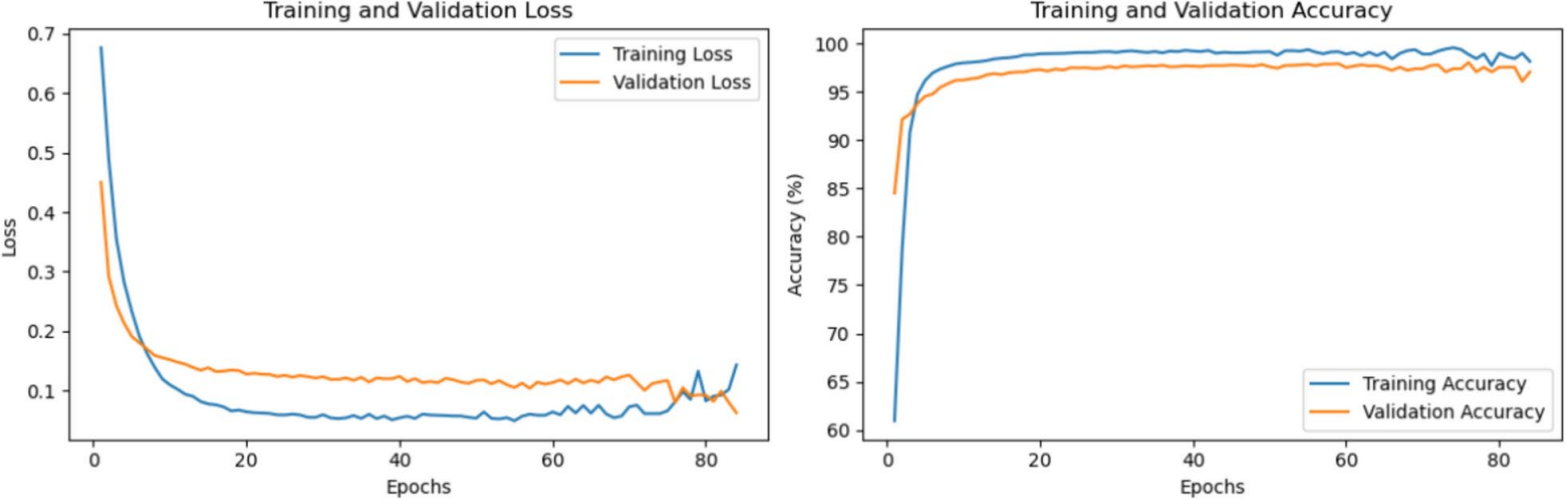
The average training and validation metrics over epochs during DS-IID training to discriminate between normal and abnormal users.

The model was trained on a dataset to differentiate between normal user profiles and those exhibiting malicious behaviour (Figure 3). To account for randomness, the evaluation of the model was conducted 100 times, and the mean evaluation scores were subsequently calculated as presented in Table 3. The model achieved a respectable accuracy of 97.3% and a Cohen’s kappa score of 0.81, indicating strong agreement beyond chance. The true positive rate (TPR) was 0.85, and the false positive rate (FPR) was 0.018, while the false acceptance rate (FAR) stood at 0.15. These metrics suggest a balanced performance between recall (0.85) and precision (0.80), culminating in an F1 score of 0.82. Notably, the AUC value was 0.99, suggesting that selecting a more optimal threshold than the default 0.5 cutoff for the binary classifier could potentially enhance these results further.

**Table 3:** The mean evaluation metrics were derived from running the model 100 times to discriminate between normal and abnormal user profiles. This rigorous testing approach ensured the reliability of the performance indicators by accounting for variability

Accuracy	Cohen’s kappa	TPR	FPR	FAR	Recall	Precision	F1 score	AUC
97.31%	0.81	0.86	0.019	0.14	0.86	0.79	0.82	0.99

### Producing synthetic user profiles

Our approach involved employing SDV’s ready-made implementations of diverse ML- and DL-based modelling techniques. Initially, we devised a schema for the real user profiles that we intended to imitate (Table S3). Utilizing this schema and the corresponding data, we trained generative models to produce analogous data. Then, the trained generative models were used to produce 1000 synthetic user profiles, comparable to the 1000 real users we already have. The synthesized data was then assessed using several evaluation metrics.

A Data Validity score is available within the SDV library. It is determined based on fundamental validity checks for each column, ensuring primary keys are always unique and non-null. It also verifies that continuous values in the synthetic data fell within the min/max range of the real data and that discrete values match the categories of the real data. Another SDV metric evaluates the data structure, confirming that the real and synthetic data share identical column names.

Additionally, two SDV scores are calculated to estimate the statistical similarity between the real and synthetic data. The first score, “Column Shapes” , measures the statistical similarity for individual columns, reflecting the marginal distribution of each column. The second score, “Column Pair Trends”, assesses the statistical similarity for pairs of columns, capturing correlations or bivariate distributions. High scores in both “Column Shapes” and “Column Pair Trends” indicate a strong alignment between the statistical properties of the real and synthetic data.

Furthermore, another set of evaluation metrics involved training our binary classification model to differentiate between real and synthesized data. The worst model results indicate a higher quality of synthetic data, as it meant the model could not easily distinguish between the two. This method serves as an additional indicator to compare different data generation techniques.

Our findings indicate that all tested synthesizers successfully generate valid and well-structured synthetic data. DL-based synthesizers, in particular, excel at producing data that closely resembles real datasets, as shown in Table  4. Among them, the TVAE synthesizer stands out by achieving the highest score for column shape similarity at 83.51%, whereas the CTGAN synthesizer leads in generating data with superior column pair trend similarity, scoring 85.74%. Additionally, our binary classification model consistently distinguishes between real and synthetic data. However, it encounters slightly more challenges when classifying data generated by TVAE and CTGAN, achieving accuracy rates of 99.37% and 99.92%, respectively.

**Table 4:** The evaluation metrics for the generative model were obtained by executing the training pipeline ten times. Each iteration involved training the model and then utilizing it to generate synthetic data. Following the generation of data in each run, quality metrics for the produced synthetic data were calculated. The mean of these quality metrics was then computed to provide a comprehensive assessment of the synthesizer’s performance

Synthesizer	Data Validity	Data Structure	Column Shapes	Column Pair Trends	Classifier accuracy
Gaussian Copula	100%	100%	76.13%	83.67%	100%
CTGAN	100%	100%	78.06%	85.74%	99.92%
TVAE	100%	100%	83.51%	84.18%	99.37%
Copula GAN	100%	100%	74.91%	83.95%	100%

### Discriminating synthetic and real malicious user profiles

**Fig. 4:**
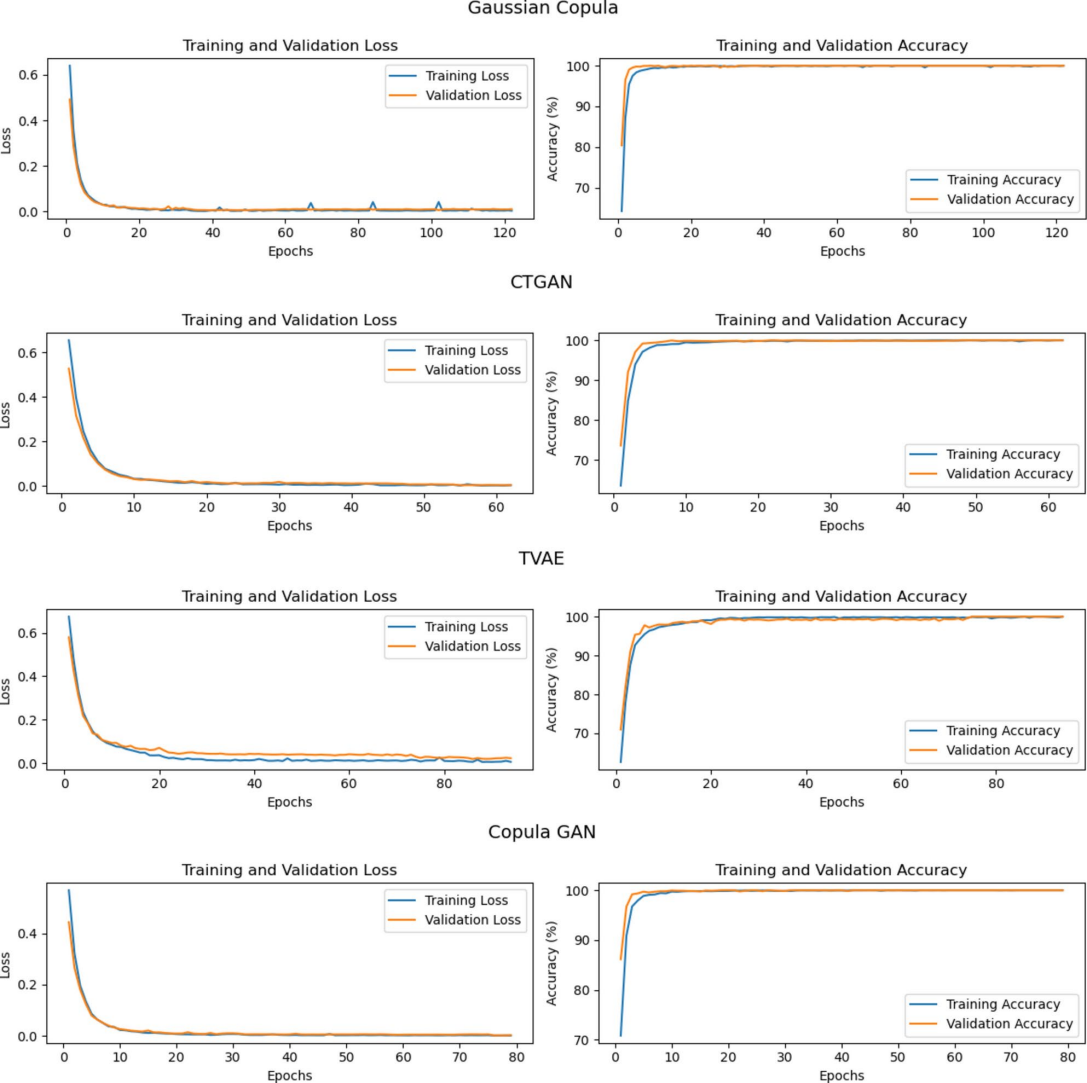
The average training and validation metrics over epochs during DS-IID training to discriminate between synthetic and real malicious user profiles.

Insider threats pose a significant challenge to detect because they originate from legitimate users. Nevertheless, detection is feasible by identifying changes in user behaviour and recognizing new patterns in data. Conversely, malicious activities from synthetic user profiles present a more complex problem for detection. Since these profiles are artificial, they may not exhibit consistent patterns in the data, making it difficult to identify deviations similar to those seen in legitimate user activity. Our binary classification model was trained to distinguish between real and synthetic data. Its ability to pinpoint malicious activities emanating from synthetic sources was then evaluated, as presented in Table  5 and Figure 4. In our evaluation, which included three distinct scenarios of abnormalities present in the data, the model maintained an accuracy rate of no less than 97% in any of the scenarios. This high level of performance highlights the model’s effectiveness in identifying and responding to a broad spectrum of user behaviours, including those that are artificially generated.

**Table 5:** The mean accuracy of training and evaluating our deep learning binary classifier ten times to distinguish real data from synthetic data produced by different sources. This evaluation encompassed all user types present in the dataset, including both normal users and those categorized as abnormal, addressing the three distinct scenarios of abnormality

Synthesizer	Normal Users	Abnormal Users	Scenario 1 Users	Scenario 2 Users	Scenario 3 Users
Gaussian Copula	100%	100%	100%	100%	100%
CTGAN	99.92%	100%	100%	100%	100%
TVAE	99.39%	98.86%	97.14%	100%	100%
Copula GAN	100%	100%	100%	100%	100%

## Discussion

In this study, we used events from the CERT dataset to build user profiles. We then applied the deep feature synthesis approach to automate features extraction from the dataset. This method eliminates the need for manual feature extraction and engineering, a typically time-consuming and intuition-driven process. By automating this step, our approach also reduces the potential for human biases that may influence the model. While domain-specific feature engineering can sometimes yield superior results, it often raises concerns about the generalizability of the solution across different contexts.

In comparison with the closest related work, Sarhan and Altwaijry  ^16^ used deep feature synthesis to automate features extraction, however, their work was carried out based on the first abnormal scenario in the CERT dataset (i.e., without considering all scenarios, discussed in Section 3.1). They also excluded two files, file.csv and email.csv, from their analysis, making their solutions unreliable. In contrast, the proposed DS-IID was tested under three abnormal scenarios in the dataset, i.e., the complete dataset. Sarhan and Altwaijry added a few manually engineered features, such as those based on normal work hours, which helped them achieve 100% accuracy using a binary SVM classifier to label user profiles as normal or abnormal. However, this overfitting was primarily due to their focus on a single scenario. Our binary deep learning model, on the other hand, achieved 97.31% accuracy, identifying malicious users while fully automating feature engineering and considering all abnormal scenarios to improve the DS-IID generalizability.

Our study also stands out for its use of different data synthesis techniques to generate synthetic data and benchmark these approaches. We found that deep learning algorithms produce synthetic user profiles that are more difficult to distinguish from real profiles compared to other machine learning algorithms. Our model, trained and tested to differentiate between synthetic and real malicious profiles, demonstrated no less than 97.14% accuracy in the worst-case scenario.

Although our introduced model is designed for offline learning, it can still be effectively utilized for real-time predictions against the latest threat vectors. The model can be integrated with data streaming frameworks and deployed in production environments to predict incoming events, thanks to the relatively small size of the neural network architecture. Additionally, periodically retraining the model with newly collected data is feasible, allowing the model to stay updated with recent trends.

## Conclusions

This paper suggested an effective and novel DS-IID model to tackle the complex challenge of insider threats in IT security, which is increasingly complicated by the use of generative AI and high-quality synthetic data. The model uses deep feature synthesis to construct accurate user profiles from event data, which significantly enhances the ability to distinguish between genuine and deceptive insider activities. The binary deep learning model employed accomplishes critical objectives including the identification of malicious users, the assessment of generative algorithms’ effectiveness at mimicking real user profiles, and the differentiation between actual and fabricated abnormal activities. The use of on-the-fly weighted random sampling to address data imbalances has proven crucial, achieving an impressive accuracy of 97% and an AUC of 0.99 in identifying malicious users. These metrics not only highlight the model’s efficiency but also its reliability in scenarios typical to insider threat detection where data is inherently imbalanced. Moreover, the experimental results showed that the model differentiates between real and synthetic malicious profiles with over 99% accuracy. One suggestion for future work is to focus on scaling this detection model and enhancing its real-world applicability, especially as generative AI technologies continue to develop. Addressing these challenges is essential for preserving the integrity of IT security systems amidst evolving technological threats.

In future work, the DS-IID model could be enhanced by incorporating a wider variety of generative models using advanced techniques such as GANs or VAEs and domain-specific feature engineering techniques. Additionally, the model could be expanded to support real-time intrusion detection by implementing streaming data processing frameworks and optimizing for low-latency predictions. Testing the model’s effectiveness across different environments on various real-world datasets is crucial allowing the model to better capture real-world complexities, such as more diverse user behaviors and environmental factors that may not be fully represented in synthetic data. Implementing explainable AI techniques could enhance its trustworthiness and usability. Continuous learning and adaptation mechanisms, such as online learning algorithms or periodic retraining with new data, could ensure that the model remains effective against the latest threat vectors.

## Supplementary Information

Supplementary Information 1.

Supplementary Information 2.

Supplementary Information 3.

## Data Availability

The dataset employed in the experiments is accessible at https://insights.sei.cmu.edu/library/insider-threat-test-dataset/ (accessed on 18 December 2023). The experiments source code is available in a GitHub repository at https://github.com/hkotb/insider-threats.
